# LncRNA LINC00483 promotes gastric cancer development through regulating MAPK1 expression by sponging miR-490-3p

**DOI:** 10.1186/s40659-020-00283-6

**Published:** 2020-04-15

**Authors:** Min Luo, Chengbai Liang

**Affiliations:** Departments of Gastroenterology, The Second Xiangya Hospital of Central South University, No. 139 RenMin Road, Changsha, 410011 Hunan China

**Keywords:** Gastric cancer, LINC00483, miR-490-3p, MAPK1

## Abstract

**Background:**

Previous studies have shown that long noncoding RNA (lncRNA) LINC00483 was aberrantly expressed in human cancers, including gastric cancer. However, the regulatory mechanism of this lncRNA in gastric cancer remains largely unknown. The present study aimed to investigate the effect of LINC00483 on gastric cancer development and explore the potential regulatory network of LINC00483/microRNA (miR)-490-3p/mitogen-activated protein kinase 1 (MAPK1).

**Methods:**

Thirty patients with gastric cancer were recruited for tissues collection. The expression levels of LINC00483, miR-490-3p and MAPK1 were detected by quantitative real-time polymerase chain reaction or western blot. Cell viability, apoptosis, migration and invasion were determined by MTT, flow cytometry, transwell assays and western blot, respectively. The target association between miR-490-3p and LINC00483 or MAPK1 was confirmed by luciferase reporter assay. Xenograft model was established to assess the function of LINC00483 in vivo.

**Results:**

LINC00483 and MAPK1 levels were increased in gastric cancer tissues and cells. Knockdown of LINC00483 or MAPK1 inhibited cells viability, migration and invasion but promoted apoptosis in gastric cancer cells. Moreover, MAPK1 overexpression attenuated the effect of LINC00483 knockdown on gastric cancer development. LINC00483 could increase MAPK1 expression by competitively sponging miR-490-3p. miR-490-3p overexpression suppressed gastric cancer development, which was abated by introduction of LINC00483. Besides, inhibition of LINC00483 decreased xenograft tumor growth by regulating miR-490-3p/MAPK1 axis.

**Conclusion:**

Knockdown of LINC00483 inhibited gastric cancer development in vitro and in vivo by increasing miR-490-3p and decreasing MAPK1, elucidating a novel mechanism for understanding the development of gastric cancer.

## Background

Gastric cancer is a serious health problem with leading cause of cancer death worldwide [1]. Recently, great advances have been gained in early diagnosis and management of gastric cancer [2, 3]. However, the prognosis and treatment of patients at advanced stage remain unsatisfactory. Therefore, it is urgent to explore novel targets for understanding the pathogenesis and treatment of gastric cancer.

Long noncoding RNAs (lncRNAs) as a class of noncoding RNAs with more than 200 nucleotides in length play essential roles in diagnosis and progression of cancers [4]. Moreover, many lncRNAs are abnormally expressed in gastric cancer [5], and they have important roles in diagnosis, prognosis and development of gastric cancer [6]. For instance, Chen et al. report that LINC01939 suppresses migration and invasion of gastric cancer by decreasing miR-17-5p and increasing early growth response 2 (EGR2) [7]. Zhang et al. suggest that LINC02532 could promote proliferation, migration and invasion of gastric cancer cells [8]. Furthermore, Hu et al. reveal that LINC00337 contributes to proliferation of gastric cancer cells by regulating p21 and enhancer of zeste homolog 2 (EZH2) [9]. As for LINC00483, a novel lncRNA, it has been indicated to promote cancer development in endometrial cancer, lung adenocarcinoma and colorectal cancer [10–12]. Besides, emerging evidence suggests that LINC00483 could facilitate proliferation, migration and invasion of gastric cancer [13]. However, little is known about the mechanism allows this lncRNA in regulation of gastric cancer development.

The available evidence indicates that lncRNA exhibits pivotal roles in regulating gene expression via functioning as competing endogenous RNA (ceRNA) of miRNA [14]. Previous studies have suggested miR-490-3p as a tumor suppressor in multiple cancers, including esophageal squamous cell carcinoma, prostate cancer and glioma [15–17]. Moreover, accruing works demonstrate the importance of miR-490-3p in gastric cancer development by acting as a tumor suppressor [18–20]. In addition, mitogen-activated protein kinase (MAPK) signaling is activated and could be as therapeutic target in human cancers [21]. In studies on gastric cancer, MAPK1 has been reported as promising target of miRNAs to participate in the development of gastric cancer [22, 23]. More importantly, the database of miRcode and TargetScan online predicted that LINC00483 and MAPK1 have the similar complementary sequences of miR-490-3p, indicating the potential ceRNA network of LINC00483/miR-490-3p/MAPK1. In this study, we explored the biological role of LINC00483 and focused on the regulatory mechanism of LINC00483 in gastric cancer. By combining in vitro and in vivo experiments, we analyzed the carcinogenic role of LINC00483 and the interaction with miR-490-3p and MAPK1 in gastric cancer.

## Materials and methods

### Patient samples and cell culture

Thirty cancer tissues and matched adjacent normal tissues were collected from patients with gastric cancer recruited from the Second Xiangya Hospital of Central South University. Patients have received radiotherapy or chemotherapy were excluded and the tissues were stored at − 80 °C. Written informed consents were provided by all participants and this study was approved by the Ethics Committee of the Second Xiangya Hospital of Central South University.

The human gastric mucosal epithelial cell GES-1 and gastric cancer cell lines (AGS, MKN-74, MKN-45 and MGC-803) were purchased from BeNa Culture Collection (Beijing, China) and cultured in DMEM (Sigma, St. Louis, MO, USA) containing 10% fetal bovine serum at 37 °C with 5% CO_2_.

### Cell transfection

The full-length sequences of LINC00483 and MAPK1 were inserted into pcDNA3.1 vector (Thermo Fisher Scientific, Wilmington, DE, USA) to generate corresponding overexpression vectors (LINC00483 and MAPK1), and pcDNA3.1 empty vectors (pcDNA) was used as a control. Small hairpin RNA (shRNA) targeted LINC00483 (sh-LINC00483-1: 5′-GGATCTAGTGAAGCCTATTCA-3′; sh-LINC00483-2: 5′-GCTTAAAGCTGCAAGCTTTCT-3′) or MAPK1 (sh-MAPK1-1: 5′-GGACCTCATGGAAACAGATCT-3′; sh-MAPK1-2: 5′-GCTGCATTCTGGCAGAAATGC-3′) were constructed by cloning into pGFP-V-RS vector and empty shRNA vector was used as negative control (sh-NC). miR-490-3p mimic (5′-CAACCUGGAGGACUCCAUGCUC-3′) and miRNA negative control (miR-NC) (5′-UUCUCCGAACGUGUCACGUTT-3′) were generated by GenePharm (Shanghai, China). Total of 30 nM oligonucleotides were used for transfection in MKN-45 and MGC-803 cells by using Lipofectamine 2000 (Thermo Fisher Scientific) for 24 h.

### 3-(4,5-Dimethyl-2-thiazolyl)-2, 5-diphenyl-2-*H*-tetrazolium bromide (MTT)

After the transfection, MKN-45 and MGC-803 cells (3 × 10^3^/well) were seeded into 96-well plates in triplicates. After incubation for 24, 48, 72 or 96 h, cells were maintained in fresh medium containing 0.5 mg/mL MTT solution (Beyotime, Shanghai, China) at 37 °C for another 4 h, followed by addition of 100 μL DMSO for solubilization of formazan. Cell viability was determined by measuring the optical density (OD) value at 490 nm through a microplate reader (Bio-Rad, Hercules, CA, USA).

### Flow cytometry

Cell apoptosis was assessed by flow cytometry using an Annexin V-FITC Apoptosis Detection Kit (Beyotime). MKN-45 and MGC-803 cells (2 × 10^5^/well) were placed into 24-well plates in triplicates and cultured for 96 h. Cells were washed with PBS, resuspend in binding buffer, and then stained with Annexin V-FITC and PI for 15 min in the dark. The cell apoptosis was analyzed with a flow cytometer (BD, San Jose, CA, USA) and apoptotic rate was expressed as percentage of cells at early and late apoptotic phases.

### Transwell assay

MKN-45 and MGC-803 cells (2 × 10^5^/well) were seeded in serum-free medium in the upper chambers with Matrigel-coated membranes for invasion assay and without coated membranes for migration assay. Meanwhile, the lower chambers were filled with 500 μL DMEM containing 10% fetal bovine serum. After a 24-h culture, the cells transferred to the membranes were stained with 0.1% crystal violet and counted under a microscope (Olympus, Tokyo, Japan) with three random fields (magnification 200×).

### Quantitative real-time polymerase chain reaction (qRT-PCR)

The RNA was extracted from gastric cancer tissues or cells using Trizol reagent (Thermo Fisher Scientific) according to the manufacturer’s instructions. After detection of concentration with a NanoDrop 2000 spectrophotometer (Thermo Fisher Scientific), 500 ng RNA was used to synthesize cDNA using Universal cDNA Synthesis Kit (Roche, Basel, Switzerland). qRT-PCR was constructed using SYBR mix (TaKaRa, Dalian, China) on ABI 7500 Real-time PCR Systems (Applied Biosystems, Foster City, CA, USA). The primer sequences used in this study were listed as: LINC00483 (Forward, 5′-GCTGAACCGGAACAGGACAT-3′; Reverse, 5′-CCAGTTCACAGCAACTCACG-3′); MAPK1 (Forward, 5′-CATGGTGTGCTCTGCTTATG-3′; Reverse, 5′-CTAGGTCTGGTGCTCAAAGG-3′); miR-490-3p (Forward, 5′-GCAAACAACCATTCGGCTGTC-3′; Reverse, 5′-CGCAGGTCCGGAGTAGGT-3′); GAPDH (Forward, 5′-GAATGGGCAGCCGTTAGGAA-3′; Reverse, 5′-AAAAGCATCACCCGGAGGAG-3′); and U6 (Forward, 5′-CTCGCTTCGGCAGCACA-3′; Reverse, 5′-TGGTGTCGTGGAGTCG-3′). GAPDH and U6 were used as endogenous controls and the relative expression levels of LINC00483, miR-490-3p and MAPK1 mRNA were analyzed by 2^−ΔΔCt^ method [24].

### Western blot

RIPA buffer (Beyotime) containing protease inhibitor was used for protein extraction from the collected tissues or cells. The protein concentration was determined by using BCA Kit (Beyotime). Then equal amounts of proteins were denatured by boiling water bath for 10 min and then separated by SDS-PAGE. The separated proteins were transferred onto 0.45 μm PVDF membranes (Millipore, Billerica, MA, USA) and then blocked with 5% non-fat milk for 1 h. Subsequently, the membranes were incubated with primary antibodies against MAPK1 (sc-136288, 1:1000 dilution, Santa Cruz Biotechnology, Santa Cruz, CA, USA), c-Myc (ab39688, 1:500 dilution, Abcam, Cambridge, MA, USA), Bax (ab199677, 1:1000 dilution, Abcam) or MMP9 (ab119906, 1:1000 dilution, Abcam) at 4 °C overnight and corresponding secondary antibody at room temperature for 2 h. GAPDH (ab37168, 1:2000 dilution, Abcam) was used as an internal control. The ECL system (Beyotime) was applied to visualize the protein blots and the relative protein levels were normalized to corresponding control group.

### Luciferase reporter assay

The potential target association between miR-490-3p and LINC00483 or MAPK1 was predicted by miRcode (http://www.mircode.org/) [25] or TargetScan (http://www.targetscan.org/vert_72/) [26]. The wild-type (WT) sequences of LINC00483 and 3′UTR sequences of MAPK1 containing complementary sites of miR-490-3p were cloned into pGL3-control luciferase reporter vectors (Promega, Madison, WI, USA) and generated WT-LINC00483 and MAPK1 3′UTR-WT respectively. Meanwhile, the mutant (MUT) vectors (MUT-LINC00483 and MAPK1 3′UTR-MUT) were generated by mutating the predicted binding sites with a Q5 Site Directed Mutagenesis Kit (New England Biolabs, Ipswich, MA, USA). MKN-45 and MGC-803 cells were co-transfected with these constructed WT or MUT luciferase reporter vectors, together with miR-490-3p or miR-NC for 24 h. Then the luciferase activity was detected using luciferase reporter system (Promega).

### Xenograft model

The procedures of this animal experiment were approved by the Ethics Committee of the Second Xiangya Hospital of Central South University. Five-week-old male BALB/c nude mice were purchased from Shanghai Animal Laboratory Center (Shanghai, China) and then randomly divided into two groups (n = 7 per group). MGC-803 cells (2 × 10^6^ cells) stably transfected with sh-LINC00483 or sh-NC were subcutaneously injected into the left flank of the mice. The tumor volume was monitored every week and calculated as volume (mm^3^) = length × width^2^ × 0.5. After cell injection for 5 weeks, mice were killed and tumor tissues were harvested. Tumor weight and related molecular analyses were determined.

### Statistical analysis

The experiments were repeated more than three times with 3 replicates and data were expressed as mean ± standard deviation (SD). Statistical analysis was performed by GraphPad Prism 7 software (GraphPad Inc., La Jolla, CA, USA) to compare the difference in different groups via Student’s *t* test or ANOVA followed by Tukey’s test. The linear relationship among the levels of LINC00483, miR-490-3p and MAPK1 in gastric cancer tissues was analyzed by spearman’s correlation coefficient. *P *< 0.05 was considered significant.

## Results

### The levels of LINC00483 and MAPK1 are increased in gastric cancer

**Fig. 1:**
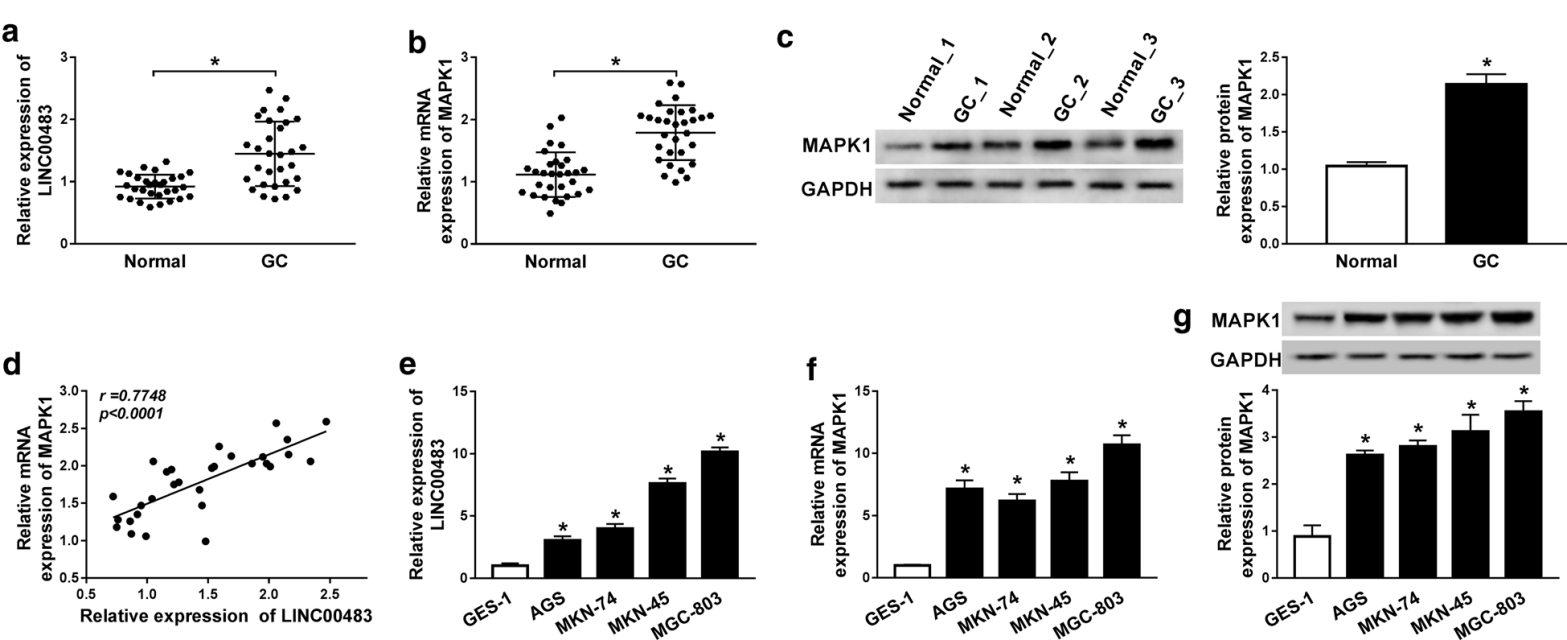
The expression levels of LINC00483 and MAPK1 are up-regulated in gastric cancer. **a**, **b** qRT-PCR assay detected the levels of LINC00483 and MAPK1 in gastric cancer tissues and normal samples. n = 30. **c** Western blot assay was performed to measure the MAPK1 protein level in gastric cancer tissues and normal tissues. **d** The association between levels of LINC00483 and MAPK1 in gastric cancer tissues was assessed. **e**–**g** The expression levels of LINC00483 and MAPK1 were detected in gastric cancer cells via qRT-PCR or western blot. GC: gastric cancer. *P < 0.05 compared with normal or GES-1 group

The expression levels of LINC00483 and MAPK1 were measured in 30 gastric cancer tissues. As shown in Fig. 1a, b, the levels of LINC00483 and MAPK1 mRNA were markedly enhanced in gastric cancer tissues compared with those in adjacent normal samples. Meanwhile, the protein expression of MAPK1 was also notably up-regulated in gastric cancer tissues in comparison to that in normal group (Fig. 1c). Moreover, there was a positive correlation between levels of MAPK1 and LINC00483 in gastric cancer tissues (r = 0.7748, *P *< 0.0001) (Fig. 1d). In addition, their abundances were also examined in gastric cancer cells. Compared with GES-1 cells, the levels of LINC00483 and MAPK1 mRNA and protein were significantly increased in gastric cancer cells (AGS, MKN-74, MKN-45 and MGC-803 (Fig. 1e–g). MKN-45 and MGC-803 cells with relative higher expression of LINC00483 were used for further experiments.

### Knockdown of LINC00483 suppresses progression of gastric cancer cells

**Fig. 2:**
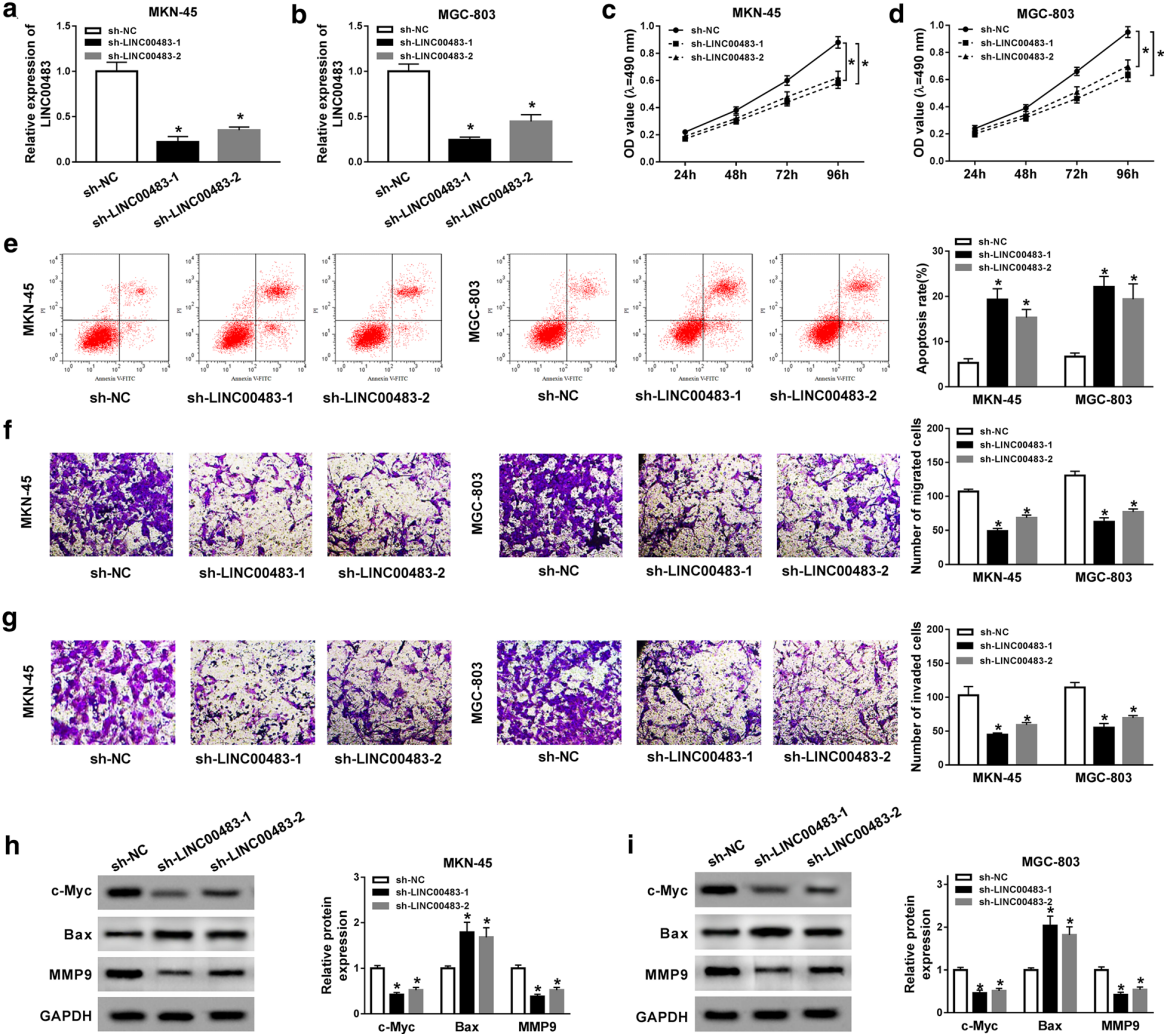
Knockdown of LINC00483 inhibits cell viability, migration and invasion but promotes apoptosis in gastric cancer cells. **a**, **b** qRT-PCR assay was performed to analyze the transfection efficacy in MKN-45 and MGC-803 cells after transfection of sh-LINC00483-1, sh-LINC00483-2 or sh-NC. **c**, **d** Cell viability was measured in MKN-45 and MGC-803 cells transfected with sh-LINC00483-1, sh-LINC00483-2 or sh-NC by MTT. **e** Cell apoptosis was detected in MKN-45 and MGC-803 cells transfected with sh-LINC00483-1, sh-LINC00483-2 or sh-NC by flow cytometry. **f**, **g** Cell migration and invasion were determined in MKN-45 and MGC-803 cells transfected with sh-LINC00483-1, sh-LINC00483-2 or sh-NC by transwell assay. **h**, **i** The protein levels of c-Myc, Bax and MMP9 were measured in MKN-45 and MGC-803 cells transfected with sh-LINC00483-1, sh-LINC00483-2 or sh-NC by western blot. sh-LINC00483: LINC00483 knockdown using shRNA; sh-NC: shRNA negative control. *P < 0.05 compared with sh-NC group

To investigate the effect of LINC00483 on gastric cancer development, its abundance was knocked down in MKN-45 and MGC-803 cells using sh-LINC00483-1 and sh-LINC00483-2. The transfection efficacy was confirmed in Fig. 2a, b. Moreover, the data of MTT assay showed that knockdown of LINC00483 evidently decreased viability of MKN-45 and MGC-803 cells at 96 h (Fig. 2c, d). In addition, down-regulation of LINC00483 led to great apoptosis in MKN-45 and MGC-803 cells at 96 h (Fig. 2e). Furthermore, the abilities of migration and invasion in MKN-45 and MGC-803 cells were significantly repressed by interference of LLINC00483 (Fig. 2f, g). Besides, the levels of protein associated with these processes were detected. Results displayed that knockdown of LINC00483 led to obvious reduction of c-Myc and MMP9 protein levels and increase of Bax level in the two cell lines (Fig. 2 h, i).

### Silence of MAPK1 inhibits progression of gastric cancer cells

**Fig. 3:**
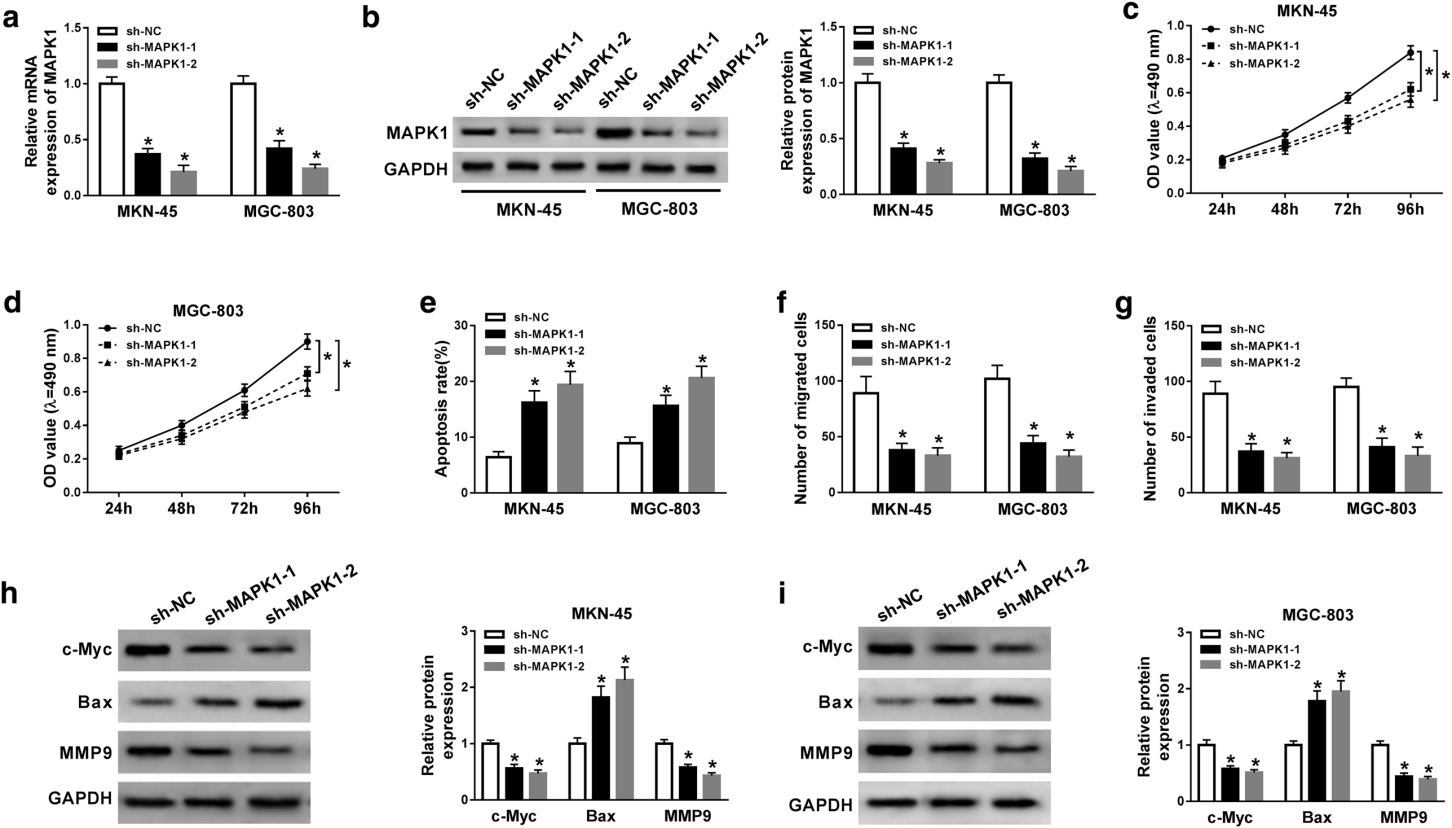
Interference of MAPK1 suppresses cell viability, migration and invasion but induces apoptosis in gastric cancer cells. **a**, **b** The abundances of MAPK1 mRNA and protein in MKN-45 and MGC-803 cells were measured after transfection of sh-MAPK1-1, sh-MAPK1-2 or sh-NC via qRT-PCR and western blot. Cell viability (**c**, **d**), apoptosis (**e**), migration (**f**), invasion (**g**) and related protein levels (**h**, **i**) were determined in MKN-45 and MGC-803 cells transfected with sh-MAPK1-1, sh-MAPK1-2 or sh-NC via MTT, flow cytometry, transwell assay and western blot. sh-MAPK1: MAPK1 knockdown using shRNA; sh-NC: shRNA negative control. *P < 0.05 compared with sh-NC group

The role of MAPK1 in gastric cancer development was evaluated in MKN-45 and MGC-803 cells transfected with sh-MAPK1-1, sh-MAPK1-2 or sh-NC. The expression of MAPK1 was effectively decreased at mRNA and protein levels in MKN-45 and MGC-803 cells transfected with sh-MAPK1-1 or sh-MAPK1-2 compared with that in sh-NC group (Fig. 3a, b). Furthermore, results showed that viability of MKN-45 and MGC-803 cells was significantly reduced by knockdown of MAPK1 at 96 h (Fig. 3c, d). Meanwhile, inhibition of MAPK1 induced higher apoptotic rate in MKN-45 and MGC-803 cells at 96 h (Fig. 3e, Additional file 1: Figure S1A). What’s more, transwell analysis described that the number of migrated and invasive cells was remarkably smaller in sh-MAPK1-1 and sh-MAPK1-2 group than that in sh-NC group (Fig. 3f, g, Additional file 1: Figure S1B and 1C). Besides, the protein levels of c-Myc and MMP9 were significantly decreased and Bax level was increased by knockdown of MAPK1 in MKN-45 and MGC-803 cells (Fig. 3h, i).

### Restoration of MAPK1 reverses the effect of LINC00483 knockdown on progression of gastric cancer cells

**Fig. 4:**
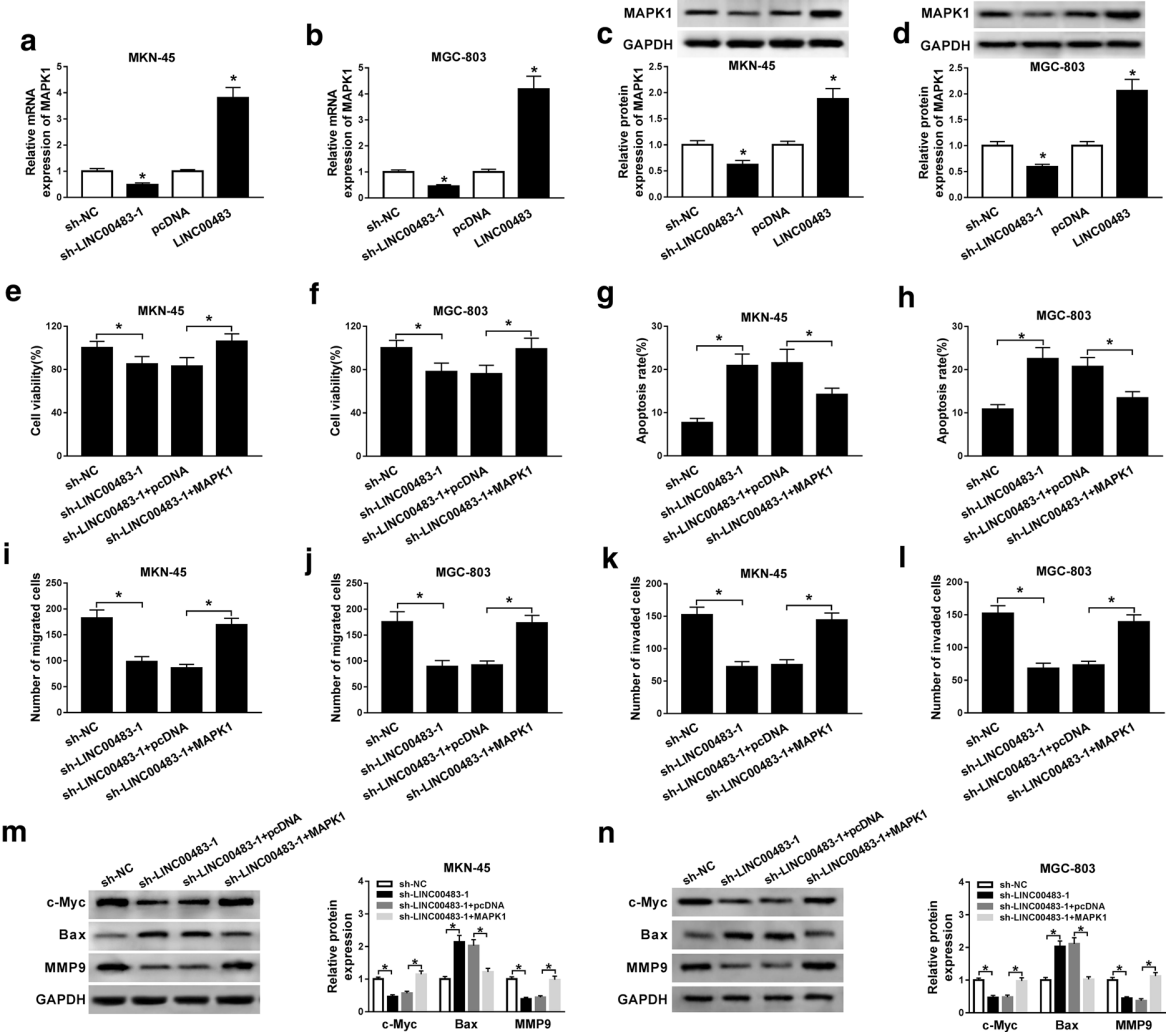
MAPK1 overexpression reverses the effect of LINC00483 knockdown on cell proliferation, apoptosis, migration and invasion in gastric cancer cells. **a**–**d** The mRNA and protein levels of MAPK1 were determined in MKN-45 and MGC-803 cells transfected with sh-LINC00483-1, sh-NC, LINC00483 or pcDNA via qRT-PCR and western blot. Cell viability (**e**, **f**), apoptosis (**g**, **h**), migration (**i**, **j**), invasion (**k**, **l**) and related protein levels (M and N) were examined in MKN-45 and MGC-803 cells transfected with sh-NC, sh-LINC00483-1, sh-LINC00483-1 and pcDNA or MAPK1 via MTT, flow cytometry, transwell assay and western blot. sh-LINC00483-1: LINC00483 knockdown using shRNA; sh-NC: shRNA negative control; LINC00483: LINC00483 overexpression; MAPK1: LINC00483; pcDNA: empty vector for overexpressing genes. *P < 0.05 compared with matched group

As shown in Fig. 4a–d, the expression of MAPK1 in MKN-45 and MGC-803 cells was positively regulated by LINC00483. In order to explore whether MAPK1 was required for LINC00483-mediated regulation on gastric cancer progression, MKN-45 and MGC-803 cells were transfected with sh-NC, sh-LINC00483, sh-LINC00483 + pcDNA or MAPK1. As displayed in Fig. 4e, f, introduction of MAPK1 restored the viability of MKN-45 and MGC-803 cells inhibited by LINC00483 knockdown. Moreover, restoration of MAPK1 attenuated silence of LINC00483-induced apoptosis (Fig. 4g, h, Additional file 2: Figure S2A). Additionally, overexpression of MAPK1 weakened the suppressive effect of LINC00483 on migration and invasion in MKN-45 and MGC-803 cells (Fig. 4i–l, Additional file 2: Figure S2B, C). Besides, the regulatory effect of LINC00483 on protein levels of c-Myc, Bax and MMP9 was abated by addition of MAPK1 in the two cell lines (Fig. 4m, n).

### LINC00483 positively regulated MAPK1 by sponging miR-490-3p in gastric cancer cells

**Fig. 5:**
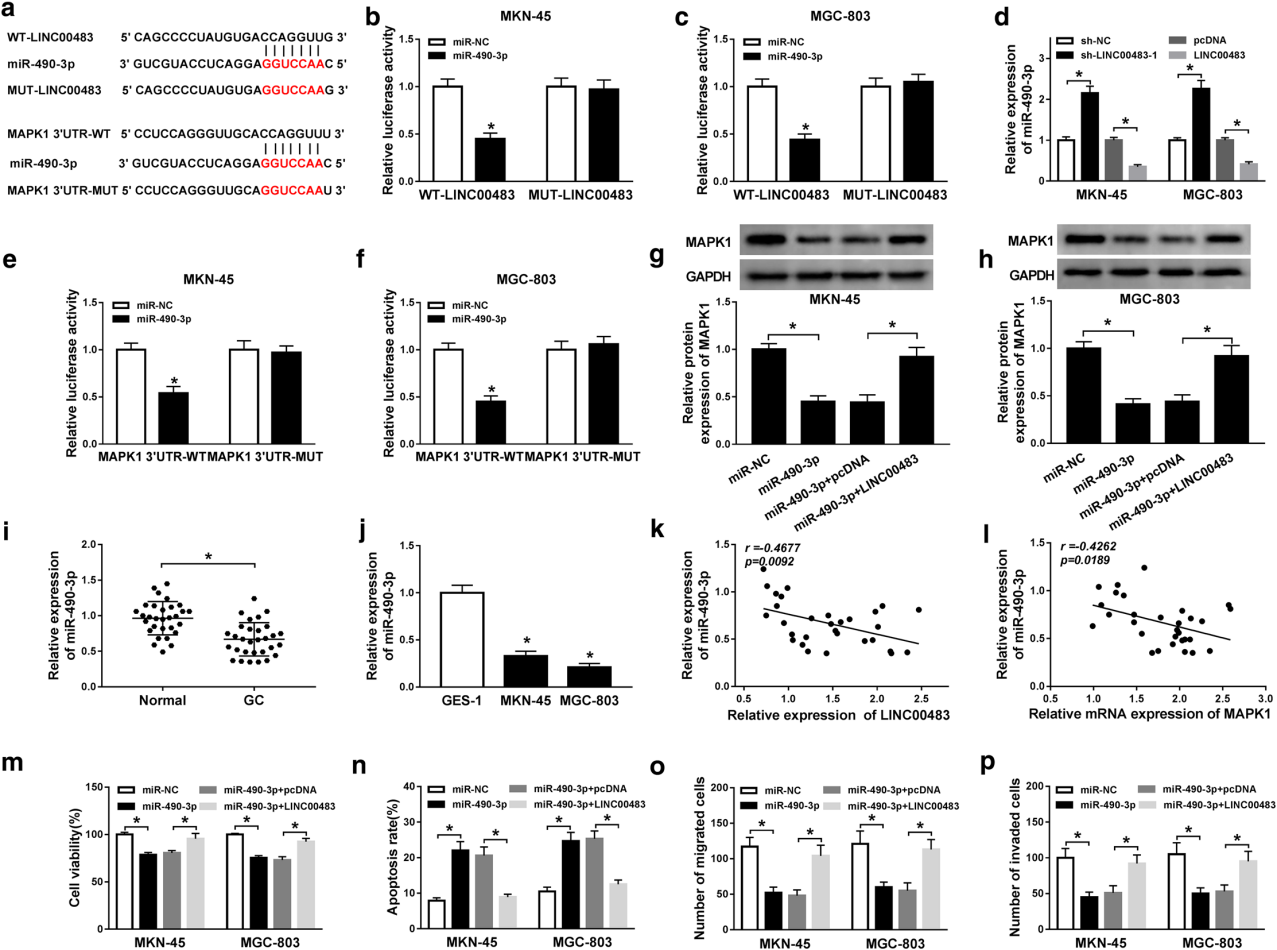
LINC00483 sponges miR-490-3p to regulate MAPK1 expression in gastric cancer cells. **a** The complementary sequences between miR-490-3p and LINC00483 or MAPK1 were predicted by miRcode or TargetScan. **b**, **c** Luciferase activity was measured in MKN-45 and MGC-803 cells co-transfected with WT-LINC00483 or MUT-LINC00483 and miR-490-3p or miR-NC. **d** The effect of LINC00483 on miR-490-3p level in MKN-45 and MGC-803 cells was investigated. **e**, **f** Luciferase reporter assay was performed in MKN-45 and MGC-803 cells co-transfected with MAPK1 3′UTR-WT or MAPK1 3′UTR-MUT and miR-490-3p or miR-NC. **g**, **h** The regulatory effect of miR-490-3p and LINC00483 on MAPK1 protein level was assessed in MKN-45 and MGC-803 cells. **i**, **j** The expression of miR-490-3p was detected in gastric cancer tissues (n = 30) and cells. **k**, **l** The association of levels of miR-490-3p and LINC00483 or MAPK1 in gastric cancer tissues was analyzed. Cell viability (**m**), apoptosis (**n**), migration (**o**) and invasion (**p**) were measured in MKN-45 and MGC-803 cells transfected with miR-NC, miR-490-3p, miR-490-3p and pcDNA or LINC00483 via MTT, flow cytometry and transwell assay. WT: wild-type; MUT: mutant; miR-490-3p: miR-490-3p mimic; miR-NC: miRNA negative control; LINC00483: LINC00483 overexpression; pcDNA: empty vector for overexpressing genes. *P < 0.05 compared with matched group

To explore how LINC00483 regulated MAPK1, the bioinformatics analysis was performed. This study using miRcode and TargetScan predicted that LINC00483 and MAPK1 have the similar binding sites of multiple miRNAs. We selected 5 lowly-expressed miRNAs in gastric cancer and measured the effect of LINC00483 on their expression. In these 5 miRNAs, miR-490-3p expression was increased most by LINC00483 knockdown (Additional file 3: Figure S3). Hence, miR-490-3p was chosen for further experiments. The binding sites of miR-490-3p and LINC00483/MAPK1 were shown in Fig. 5a. To confirm the association between LINC00483 and miR-490-3p, the luciferase reporter vectors WT-LINC00483 and MUT-LINC00483 were generated and transfected into MKN-45 and MGC-803 cells. As shown in Fig. 5b, c, overexpression of miR-490-3p significantly reduced luciferase activity in WT-LINC00483 group, while it did not affect the activity in MUT-LINC00483 group. Moreover, qRT-PCR assay showed that miR-490-3p expression was negatively regulated by LINC00483 in MKN-45 and MGC-803 cells (Fig. 5d). To validate the target association between miR-490-3p and MAPK1, we constructed MAPK1 3′UTR-WT and MAPK1 3′UTR-MUT luciferase-expressing vectors and transfected them into MKN-45 and MGC-803 cells. Results displayed that luciferase activity in MKN-45 and MGC-803 cells was obviously decreased by miR-490-3p overexpression in MAPK1 3′UTR-WT group, whereas it was not changed in MAPK1 3′UTR-MUT group (Fig. 5e, f). Furthermore, analysis of western blot demonstrated that the protein level of MAPK1 was markedly decreased via miR-490-3p overexpression in MKN-45 and MGC-803 cells and this effect was abrogated by introduction of LINC00483 (Fig. 5g, h). In addition, the expression of miR-490-3p was remarkably down-regulated in gastric cancer tissues and cells when compared with normal tissues or GES-1 cells (Fig. 5i, j). What’s more, the abundance of miR-490-3p in cancer tissues was negative correlated with level of LINC00483 or MAPK1 (Fig. 5k, l). Besides, overexpression of miR-490-3p resulted in obvious reduction in viability, migration and invasion as well as increase in apoptosis of MKN-45 and MGC-803 cells, while these events were mitigated by addition of LINC00483 (Fig. 5m–p, Additional file 4: Figure S4A–C).

### Knockdown of LINC00483 decreases tumor growth in gastric cancer xenograft model

**Fig. 6:**
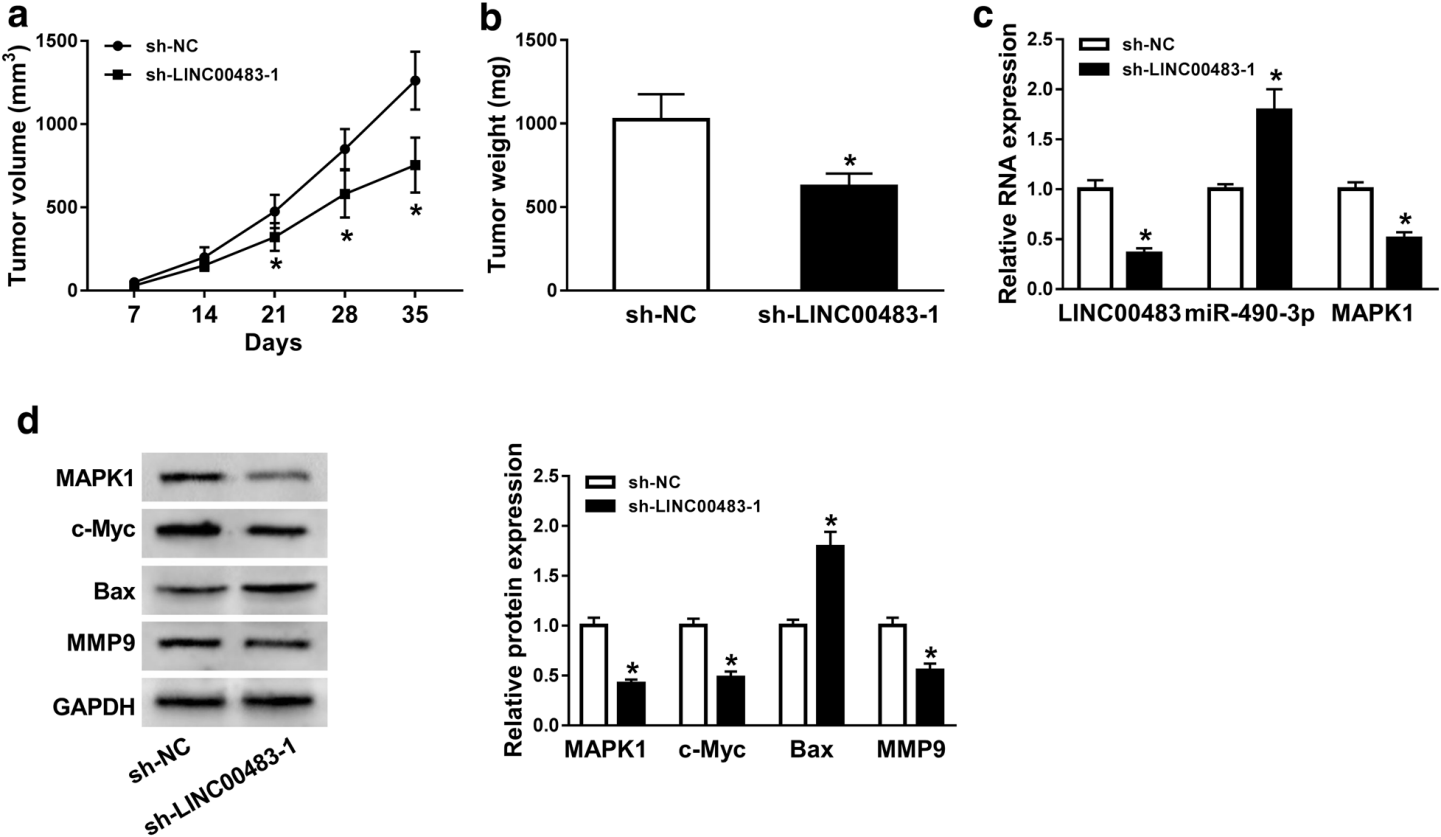
Knockdown of LINC00483 decreases tumor growth in gastric cancer xenograft model. **a** Tumor volume was measured every week. **b** Tumor weight was detected at the end. **c** qRT-PCR assay detected the expression levels of LINC00483, miR-490-3p and MAPK1 in tumor tissues. **d** Western blot assay examined the protein levels of MAPK1, c-Myc, Bax and MMP-9 in tumor tissues. sh-LINC00483: xenograft tumor formed using MGC-803 cells with LINC00483 knockdown using shRNA; sh-NC: xenograft tumor formed using MGC-803 cells with transfection of shRNA negative control. *P < 0.05 compared with sh-NC group

In order to evaluate the role of LINC00483 in gastric cancer in vivo, MGC-803 cells stably transfected with sh-LINC00483 or sh-NC were injected into nude mice and classified as sh-LINC00483 and sh-NC group, respectively. As shown in Fig. 6a, b, the tumor volume and weight were markedly decreased in sh-LINC00483 group compared with those in sh-NC group. Moreover, the data of qRT-PCR assay showed that the level of miR-490-3p was significantly increased in sh-LINC00483 group compared with that in sh-NC group, while the abundances of LINC00483 and MAPK1 displayed opposite trend (Fig. 6c). Additionally, results of western blot presented that the protein levels of MAPK1, c-Myc and MMP9 in tumor tissues were significantly reduced but Bax expression was enhanced in sh-LINC00483 group in comparison to those in sh-NC group (Fig. 6d).

## Discussion

LncRNAs are abnormally expressed and implicated in regulation of cell processes in gastric cancer [27]. LINC00483 as a novel lncRNA has been reported as key oncogene in human cancers, including gastric cancer [11–13]. Previous study has reported that LINC00483 expression was enhanced and promoted gastric cancer cell proliferation via regulating miR-30a-3p and sperm-associated antigen 9 (SPAG9) [13]. However, the regulatory mechanism by which LINC00483 mediated gastric cancer progression remains largely unclear. This study investigated the function of LINC00483 in gastric cancer development in vitro and in vivo. The novelty of this study was MAPK1 as a novel target for LINC00483, and here we were the first to confirm LINC00483 could target MAPK1 by miR-490-3p in gastric cancer.

Here we found that LINC00483 expression was increased in gastric cancer, indicating that high expression of LINC00483 might contribute to gastric cancer development. To explore the role of LINC00483, loss-of-function experiments were performed. c-Myc is a key protein associated with metabolism, proliferation and oncogenesis of cancers [28], which could be modulated by lncRNA to influence cell viability [29]. Bax is an important member of Bcl-2 family, which displays pro-apoptotic role in cancers through intrinsic apoptosis pathway [30]. Moreover, MMP9 has been regarded as an essential marker for metastasis of cancers, including gastric cancer [31, 32]. Through detecting protein levels of c-Myc, Bax and MMP9 combined with corresponding analyses of MTT, flow cytometry and transwell, we found that LINC00483 knockdown suppressed gastric cancer development by inhibiting cell viability, migration and invasion and inducing apoptosis. These findings indicated the suppressive effect of LINC00483 inhibition on gastric cancer cell development, which was also consistent with former work [13]. This study indicated the potential anti-cancer role of LINC00483 knockdown in gastric cancer, which might be important target for treatment of gastric cancer.

MAPK1 has been reported as an important oncogene in gastric cancer progression to promote cell proliferation, migration and invasion [22, 23, 33, 34]. In this research, we also found high expression of MAPK1 in gastric cancer and its knockdown inhibited gastric cancer cell viability, migration and invasion but promoted apoptosis, indicating the carcinogenic role of MAPK1 in gastric cancer. Moreover, analysis of linear relationship showed that MAPK1 level was positively correlated with LINC00483 expression, uncovering the potential interaction between LINC00483 and MAPK1. By transfection of MAPK1 overexpression vector in the presence of sh-LINC00483, the results revealed that MAPK1 was responsible for the function of LINC00483 in gastric cancer. However, how LINC00483 could mediate MAPK1 remains unclear.

The interaction between lncRNA and RNA could be mediated by miRNA, in which lncRNA acts as a miRNA sponge for miRNA inhibition, therefore leading to derepress of mRNA [35]. To find out the potential intermediator, we performed bioinformatics analysis and found that LINC00483 and MAPK1 have the similar seed sites of miR-490-3p. Although the target association between miR-490-3p and MAPK1 has been reported by previous studies in esophageal squamous cell carcinoma and acute myeloid leukemia [36, 37], it did not indicate this axis was present in gastric cancer because of the alteration of tumor microenvironment. Here we confirmed their association using luciferase reporter assay and demonstrated that LINC00483 could up-regulate MAPK1 expression by competitively sponging miR-490-3p in gastric cancer. Moreover, there are many binding sites of LINC00483 and other miRNAs or mRNAs, such as miR-30a-3p and SPAG9 [13]. Hence, we hypothesized it was possible LINC00483 had additional targets. In any case, MAPK1 was one important target of LINC00483 in this study and it was indirectly regulated via LINC00483 through miR-490-3p. Furthermore, this study showed that miR-490-3p expression was decreased and its overexpression suppressed development of gastric cancer, which was also in agreement with previous studies [19, 20]. Additionally, rescue experiments further validated miR-490-3p was required for LINC00483-mediated regulatory mechanism in gastric cancer progression in vitro. Meanwhile, we also using a xenograft model disclosed the anti-cancer role of LINC00483 inhibition in gastric cancer.

## Conclusion

Our research on the oncogenic role of LINC00483 in gastric cancer showed that silence of LINC00483 repressed progression of gastric cancer in vitro and in vivo, possibly by acting as a sponge of miR-490-3p to regulate MAPK1, which provided a new mechanism for development of gastric cancer and indicated a novel target for treatment of gastric cancer.

## Supplementary information

**Additional file 1: Figure S1.** MAPK1 knockdown promotes apoptosis and inhibits migration and invasion in gastric cancer cells. (A) Cell apoptosis was detected in MKN-45 and MGC-803 cells transfected with sh-MAPK1-1, sh-MAPK1-2 or sh-NC by flow cytometry. (B and C) Cell migration and invasion were measured in MKN-45 and MGC-803 cells transfected with sh-MAPK1-1, sh-MAPK1-2 or sh-NC by transwell assay.

**Additional file 2: Figure S2.** MAPK1 overexpression reverses the effect of LINC00483 knockdown on cell apoptosis, migration and invasion in gastric cancer cells. (A) Cell apoptosis was detected in MKN-45 and MGC-803 cells transfected with sh-NC, sh-LINC00483-1, sh-LINC00483-1 and pcDNA or MAPK1 by flow cytometry. (B and C) Cell migration and invasion were measured in MKN-45 and MGC-803 cells transfected with sh-NC, sh-LINC00483-1, sh-LINC00483-1 and pcDNA or MAPK1 by transwell assay.

**Additional file 3: Figure S3.** The effect of LINC00483 on 5 predicted miRNAs. miRcode and TargetScan predicted the miRNAs that have the binding sites of LINC00483 and MAPK1. 5 lowly-expressed miRNAs (miR-183, miR-101-3p, miR-128-3p, miR-129-5p and miR-490-3p) in gastric cancer were selected. The effect of LINC00483 knockdown on these miRNAs’ expression was measured in MKN-45 cells. *P < 0.05.

**Additional file 4: Figure S4.** LINC00483 sponges miR-490-3p to regulate apoptosis, migration and invasion in gastric cancer cells. (A) Cell apoptosis was detected in MKN-45 and MGC-803 cells transfected with miR-NC, miR-490-3p, miR-490-3p and pcDNA or LINC00483 by flow cytometry. (B and C) Cell migration and invasion were measured in MKN-45 and MGC-803 cells transfected with miR-NC, miR-490-3p, miR-490-3p and pcDNA or LINC00483 by transwell assay.

## Data Availability

The datasets used and/or analyzed during the current study are available from the corresponding author on reasonable request.

## Abbreviations

lncRNA: Long noncoding RNA
MAPK1: Mitogen-activated protein kinase 1
EZH2: Enhancer of zeste homolog 2
EGR2: Early growth response 2
ceRNA: competing endogenous RNA
