# Supplementary figures and images for: LncRNA LINC00483 promotes gastric cancer development through regulating MAPK1 expression by sponging miR-490-3p
Source: Biol Res. 2020 Apr 15;53:14. doi: 10.1186/s40659-020-00283-6 (PMC7158027; doi:10.1186/s40659-020-00283-6)

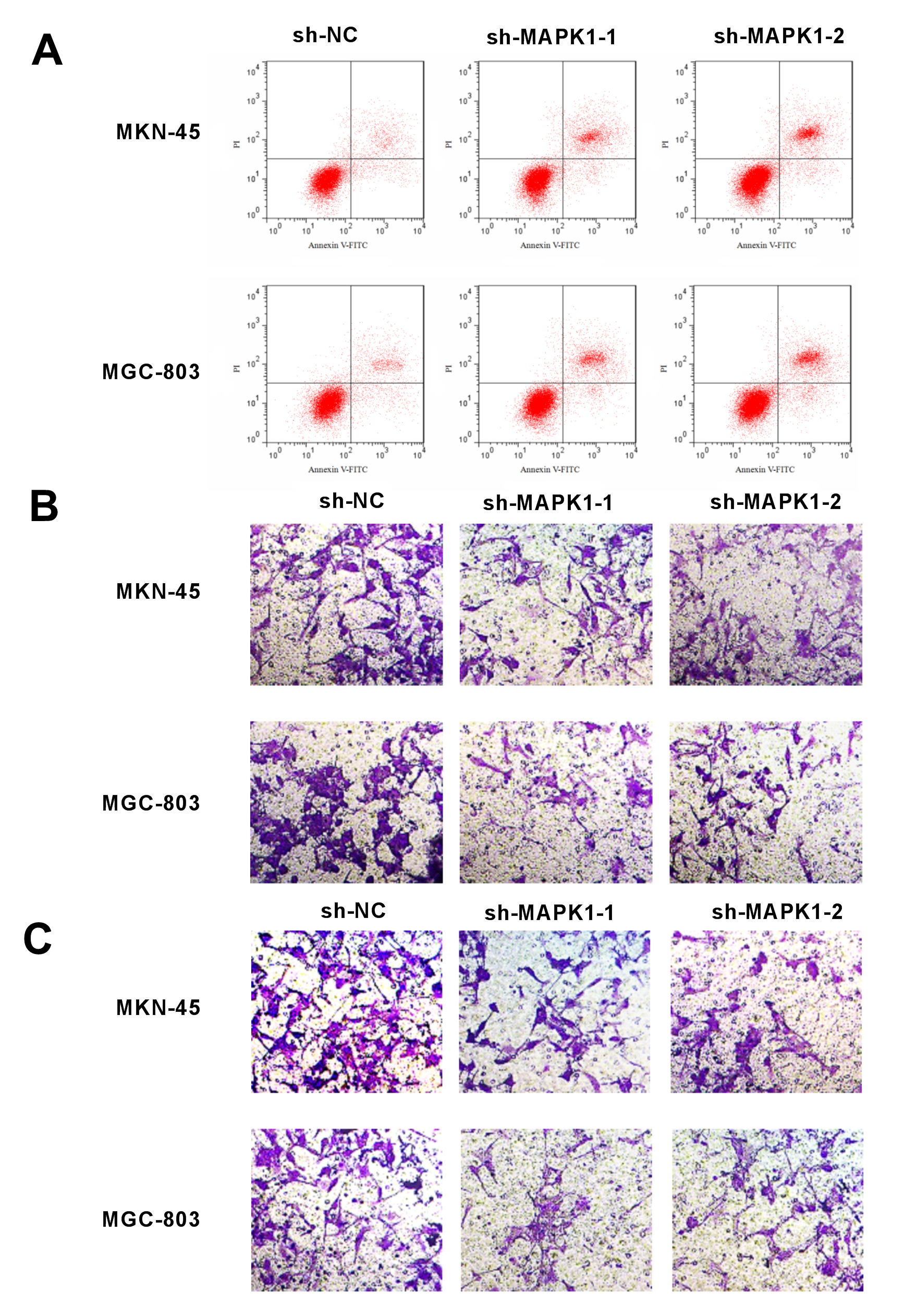

Supplement: Supplementary file 1 — Additional file 1: Figure S1. MAPK1 knockdown promotes apoptosis and inhibits migration and invasion in gastric cancer cells. (A) Cell apoptosis was detected in MKN-45 and MGC-803 cells transfected with sh-MAPK1-1, sh-MAPK1-2 or sh-NC by flow cytometry. (B and C) Cell migration and invasion were measured in MKN-45 and MGC-803 cells transfected with sh-MAPK1-1, sh-MAPK1-2 or sh-NC by transwell assay. [file 40659_2020_283_MOESM1_ESM.tif]

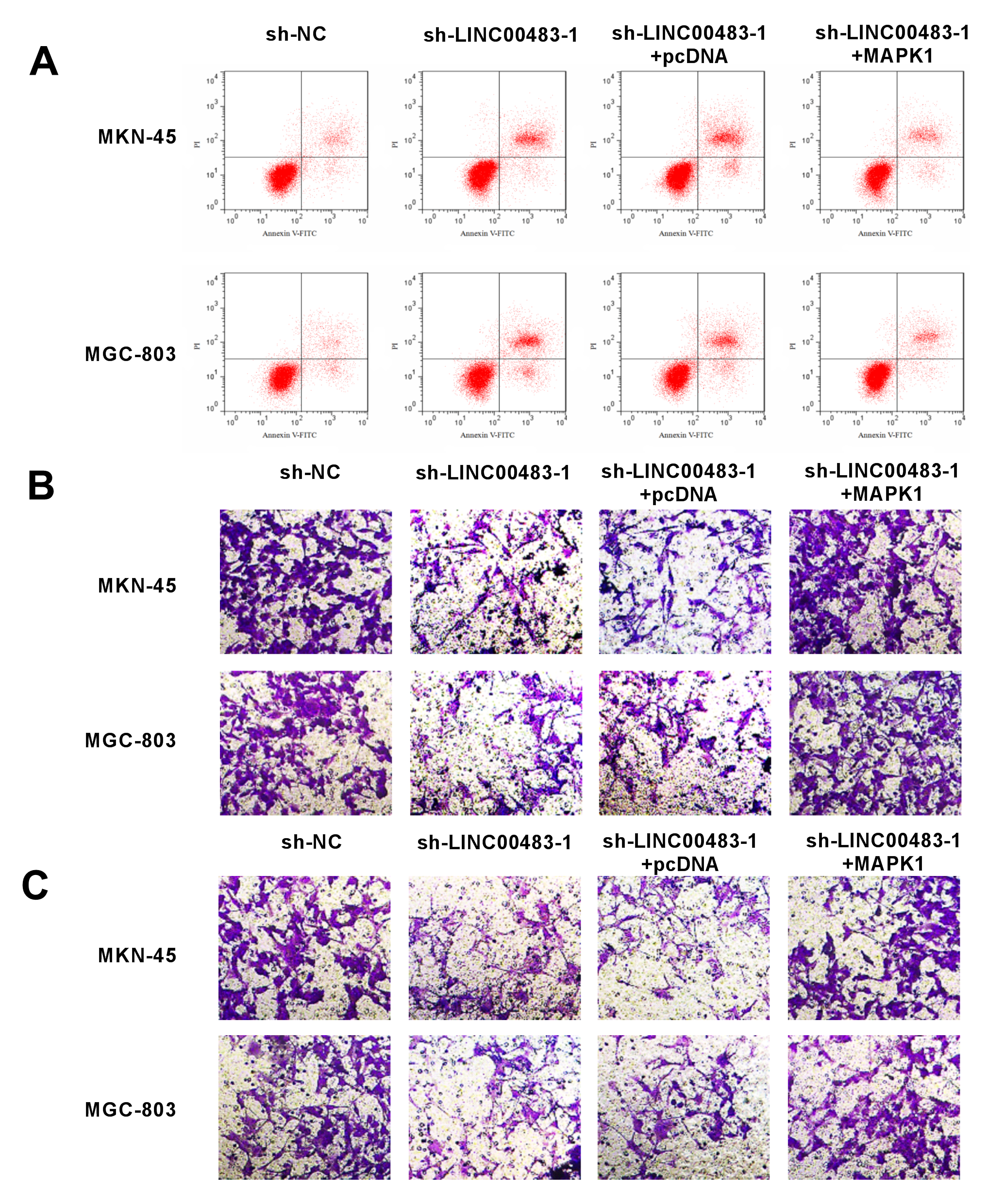

Supplement: Supplementary file 2 — Additional file 2: Figure S2. MAPK1 overexpression reverses the effect of LINC00483 knockdown on cell apoptosis, migration and invasion in gastric cancer cells. (A) Cell apoptosis was detected in MKN-45 and MGC-803 cells transfected with sh-NC, sh-LINC00483-1, sh-LINC00483-1 and pcDNA or MAPK1 by flow cytometry. (B and C) Cell migration and invasion were measured in MKN-45 and MGC-803 cells transfected with sh-NC, sh-LINC00483-1, sh-LINC00483-1 and pcDNA or MAPK1 by transwell assay. [file 40659_2020_283_MOESM2_ESM.tif]

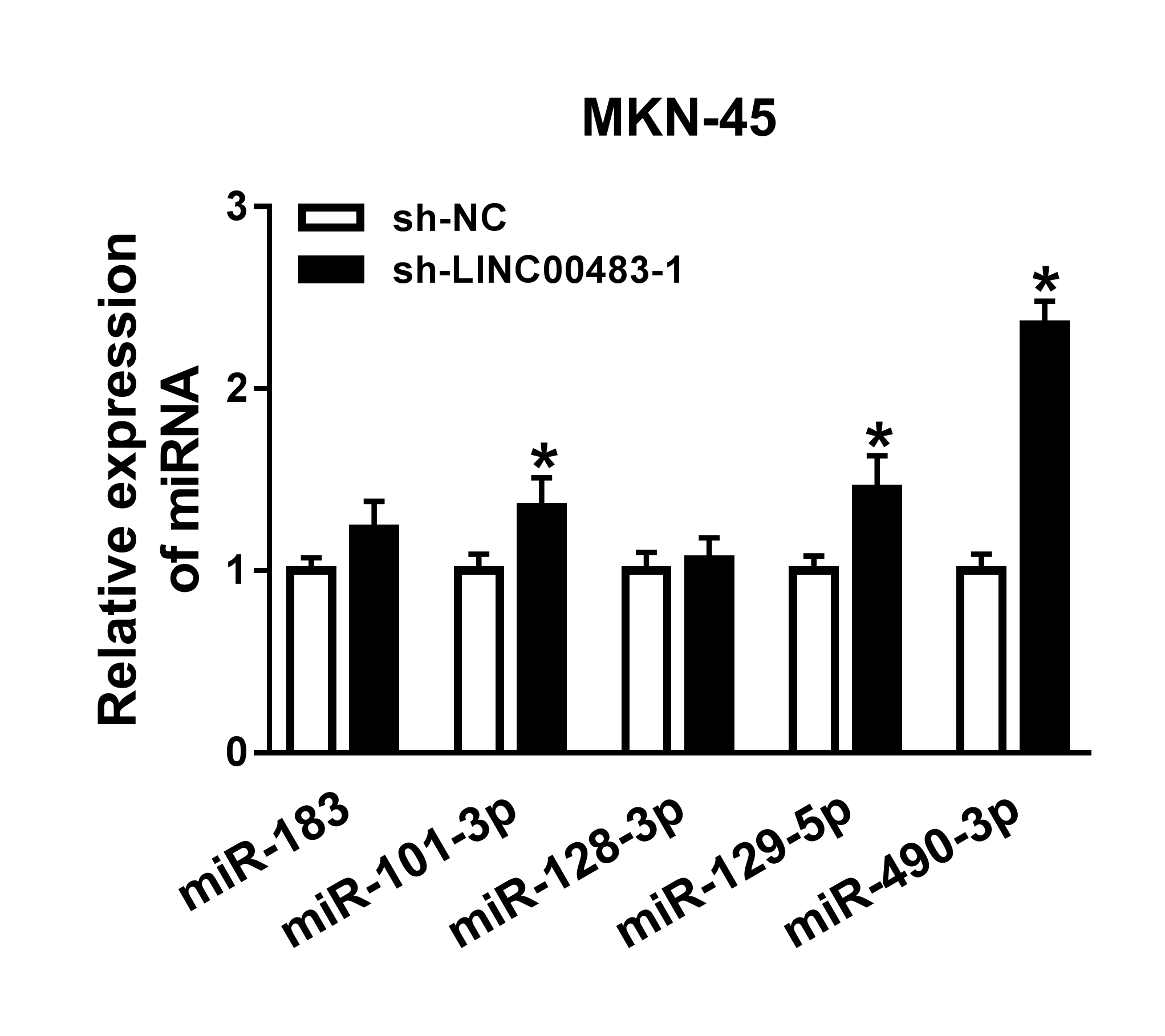

Supplement: Supplementary file 3 — Additional file 3: Figure S3. The effect of LINC00483 on 5 predicted miRNAs. miRcode and TargetScan predicted the miRNAs that have the binding sites of LINC00483 and MAPK1. 5 lowly-expressed miRNAs (miR-183, miR-101-3p, miR-128-3p, miR-129-5p and miR-490-3p) in gastric cancer were selected. The effect of LINC00483 knockdown on these miRNAs’ expression was measured in MKN-45 cells. *P < 0.05. [file 40659_2020_283_MOESM3_ESM.tif]

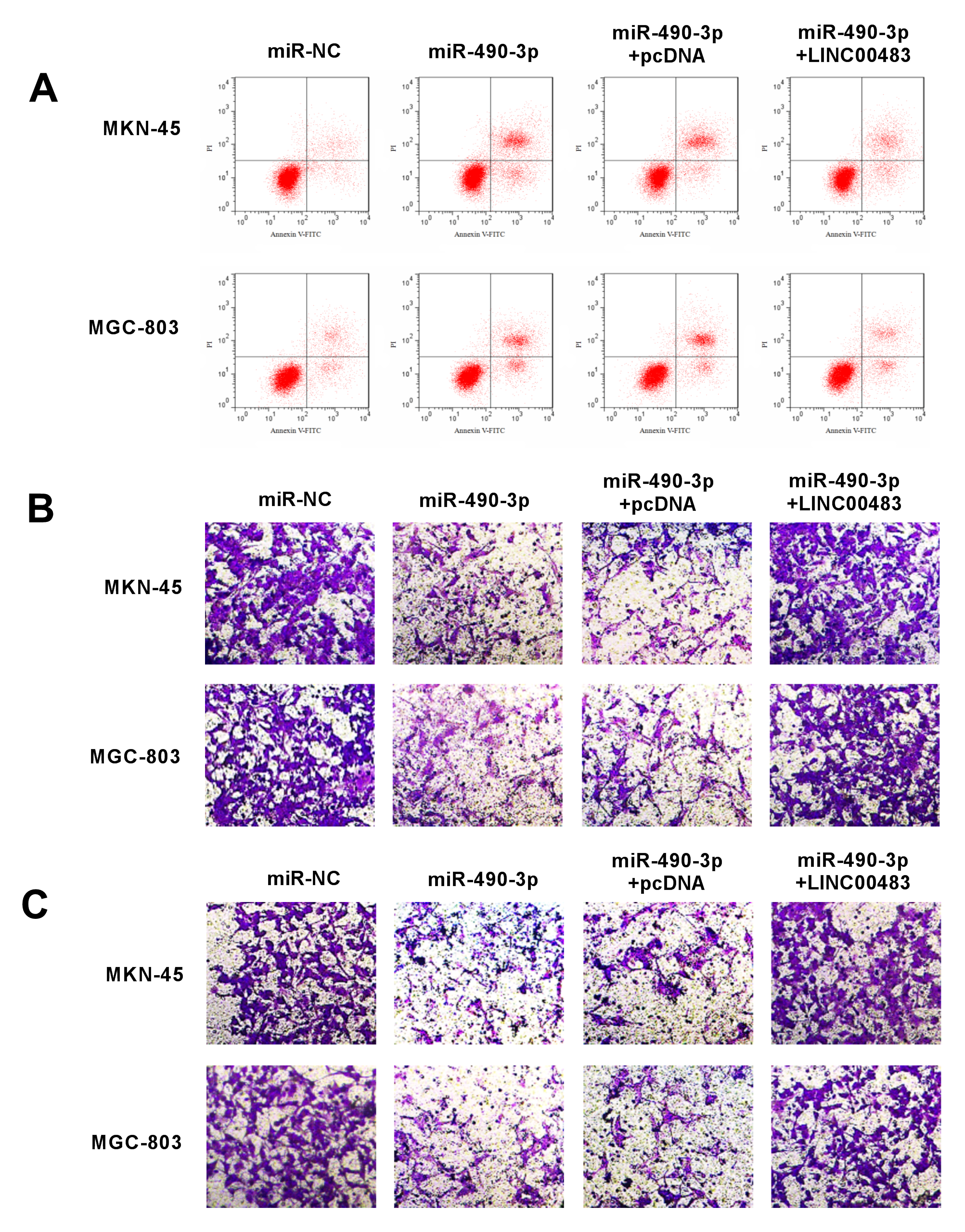

Supplement: Supplementary file 4 — Additional file 4: Figure S4. LINC00483 sponges miR-490-3p to regulate apoptosis, migration and invasion in gastric cancer cells. (A) Cell apoptosis was detected in MKN-45 and MGC-803 cells transfected with miR-NC, miR-490-3p, miR-490-3p and pcDNA or LINC00483 by flow cytometry. (B and C) Cell migration and invasion were measured in MKN-45 and MGC-803 cells transfected with miR-NC, miR-490-3p, miR-490-3p and pcDNA or LINC00483 by transwell assay. [file 40659_2020_283_MOESM4_ESM.tif]
